# Ocular Involvement in Relapsing Polychondritis

**DOI:** 10.3390/jcm10214970

**Published:** 2021-10-26

**Authors:** Ken Fukuda, Tomoka Mizobuchi, Isana Nakajima, Tatsuma Kishimoto, Yusaku Miura, Yoshinori Taniguchi

**Affiliations:** 1 Department of Ophthalmology and Visual Science, Kochi Medical School, Kochi University, Nankoku, Kochi 783-8505, Japan; jm-t.yamamoto@kochi-u.ac.jp (T.M.); jm-i-nakajima@kochi-u.ac.jp (I.N.); t.kishimoto@kochi-u.ac.jp (T.K.); miurasaku@kochi-u.ac.jp (Y.M.); 2 Department of Endocrinology, Metabolism, Nephrology and Rheumatology, Kochi Medical School Hospital, Kochi University, Nankoku, Kochi 783-8505, Japan; taniguchiy@kochi-u.ac.jp

**Keywords:** relapsing polychondritis, scleritis, uveitis, keratitis, conjunctivitis, retinopathy, optic neuropathy, ultrasonography, auricular chondritis

## Abstract

Relapsing polychondritis (RPC) is a rare systemic immune-mediated disease characterized by recurrent inflammation of cartilaginous and proteoglycan-rich tissues throughout the body. Auricular, nasal, tracheal, and articular chondritis and arthritis are common systemic symptoms in patients with RPC. Ocular tissues are also targets of inflammation in RPC, and a variety of ocular symptoms are observed in approximately half of the patients with RPC. Scleritis/episcleritis, uveitis, and conjunctivitis are common symptoms associated with RPC. Less frequently, keratitis, retinopathy, optic neuropathy, muscle palsy, and orbital inflammation are also observed. Ocular inflammation could also be the first manifestation of RPC. Although RPC is a potentially fatal and sight-threatening disease, the rarity of the disease and its protean clinical presentation may lead to delayed diagnosis or misdiagnosis. Given the high prevalence of ocular involvement in RPC, to avoid misdiagnosis, physicians should be suspicious of RPC when they see patients with recurrent ocular inflammatory conditions and various systemic symptoms. In this article, we provide a comprehensive review of ocular manifestations associated with RPC.

## 1. Introduction

Relapsing polychondritis (RPC) was first described by Jaksch-Wartenhorst [1] in 1923 as a rare systemic immune-mediated disease of unknown etiology characterized by recurrent inflammation of cartilaginous and proteoglycan-rich tissues throughout the body [2,3,4]. The incidence of RPC is estimated to be 3.5 per million population per year in the United States, and a population-based cohort study showed that the incidence of RPC between 1990 and 2012 was 0.71 per million population per year in the United Kingdom [5,6]. A recent calculated prevalence of RPC was 4.5 per million in the United States [7]. The target tissues of RPC include not only cartilaginous structures, such as the ear, nose, respiratory tract, and joints, but also non-cartilaginous tissues, such as the eyes, skin, heart, and central nervous system. Although the pathogenesis of RPC has not been fully elucidated, autoimmune reactions to type II collagen are considered essential, and both humoral and cellular immunity have been implicated in the autoimmune reactions. The rarity and wide spectrum of clinical symptoms and signs of RPC often lead to misdiagnosis or delayed diagnosis [8,9]. Given that RPC is a potentially fatal disease, prompt and correct diagnosis and treatment are essential. The eyeball and ocular adnexa are important target tissues for RPC-induced inflammation, and a wide range of ocular manifestations are observed in patients with RPC (Table 1) [10,11]. In this review, we provide an updated, comprehensive review of ocular manifestations in patients with RPC.

## 2. Ocular Manifestations of RPC

The cumulative prevalence of the ocular symptoms in RPC varies by studies, but they have been reportedly observed in 20–61% of patients with RPC (Table 2) [2,4,6,7,8,9,12,13,14,15,16,17,18]. Common ocular manifestations are scleritis, uveitis, conjunctivitis, and keratitis (Table 3). Other ocular manifestations, including eyelid edema, proptosis, retinopathy, optic neuropathy, orbital inflammation, and extraocular muscle palsy, have been reported. Ophthalmologists should be aware that ocular symptoms are one of the common initial symptoms prior to diagnosis, as shown in Table 2. A recent, large study showed that the initial symptoms were ocular symptoms, such as redness and blurred vision in 11.5% of patients with RPC [13]. In addition, ocular symptoms, such as panuveitis and keratitis [19], keratouveitis [20], retinal occlusive vasculitis [21], oculomotor and abducens nerve palsies [22], recurrent optic perineuritis [23], and orbital inflammation [24,25], have been reported as the first manifestations of RPC before systemic symptoms.

### 2.1. Sclera

**Figure 1:**
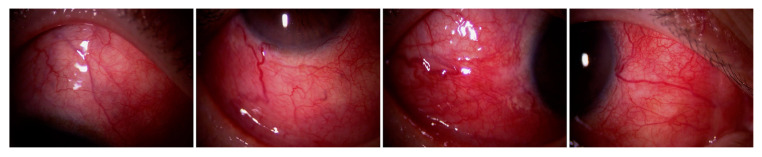
Diffuse anterior scleritis associated with RPC.

**Figure 2:**
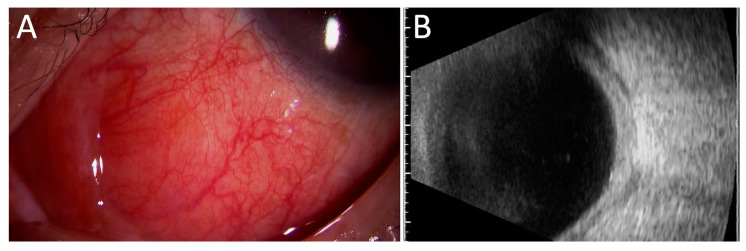
Nodular anterior and posterior scleritis associated with RPC: slit-lamp exam showed anterior nodular scleritis (**A**) and B-scan ultrasonogram (**B**) showed thickened sclera with fluid in the sub-Tenon’s space (T-sign).

Scleritis/episcleritis is the most common ocular complication associated with RPC (Table 3) [10,26,27,28,29,30,31,32,33,34]. Most cases of scleritis occur bilaterally. Several case series revealed that diffuse anterior scleritis was the most common (Figure 1), but nodular (Figure 2A) or necrotizing anterior scleritis and posterior scleritis (Figure 2B) were also observed (Table 4) [32,33,34,35]. Sainz-de-la-Maza et al. compared patients with scleritis associated with RPC and other systemic immune-mediated diseases and showed that it is more often bilateral, recurrent, necrotizing, and associated with visual disturbance than scleritis associated with other systemic immune-mediated diseases [32].

### 2.2. Uvea

**Figure 3:**
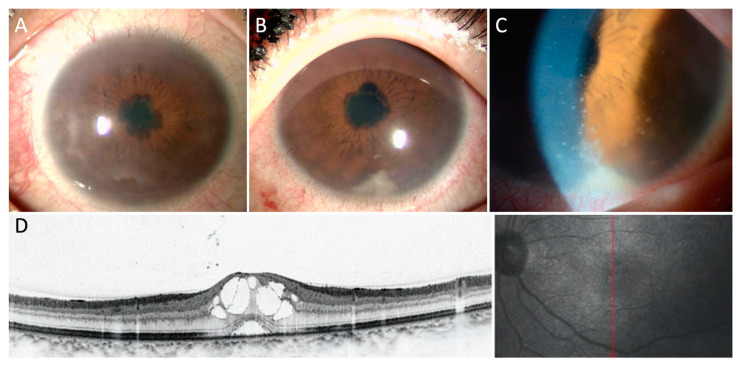
Bilateral anterior uveitis with macular edema in a patient with RPC. Photographs show bilateral anterior uveitis with posterior synechia (**A**,**B**). Keratic precipitates and hypopyon are also observed in left eye (**B**,**C**). OCT depicts cystoid macular edema (**D**).

Uveitis is also a common ocular complication in patients with RPC [2,10,33,34,36,37]; chronic anterior uveitis with hypopyon (Figure 3) is often observed [26,38,39,40,41]. In addition, panuveitis with retinitis, including retinal vasculitis and/or hemorrhage, also occurs in RPC [19,42].

### 2.3. Conjunctiva

**Figure 4:**
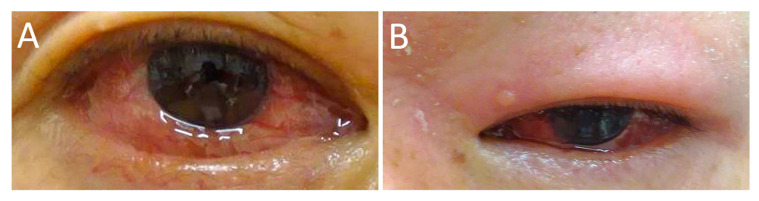
Conjunctivitis and eyelid edema in patients with RPC. Photographs show conjunctival edema and hyperemia (**A**) and swelling and redness of the upper eyelid (**B**).

Conjunctivitis (Figure 4A) with nonspecific conjunctival redness, irritation, and itching was observed in patients with RPC [2]. Subconjunctival hemorrhage and keratoconjunctivitis sicca have also been observed [10]. Chronic conjunctival inflammation reportedly results in the formation of salmon patch lesions with reactive lymphoid hyperplasia [43]. Yu et al. demonstrated the pathological examination of conjunctival biopsy [44]. Pathological examination revealed granulomatous obliterative microangiopathy with various inflammatory cells in the substantia fascia, such as eosinophils, plasma cells, lymphocytes, and epithelioid cells. Hoang-Xuan et al. also performed histologic and immunopathologic examinations of ocular biopsy specimens (three conjunctiva and one sclero-cornea) from three patients with RPC. In the conjunctiva, mast cells and chronic inflammatory cells, such as lymphocytes and plasma cells in the substantia propria of all three patients, were observed. Vasculitis was also found in the conjunctiva of two cases and sclera in one case; perivasculitis in the conjunctiva of one patient was observed. Immunofluorescent staining revealed complement (C3) and immunoglobulin (IgM and IgG) deposition in the vessel walls of conjunctiva [35].

### 2.4. Cornea

In the cornea, peripheral ulcerative keratitis is reportedly a common type of keratitis associated with RPC [10,45,46,47,48], which is similar to other connective tissue diseases, such as rheumatoid arthritis [49]. Ulceration sometimes progresses rapidly, resulting in corneal melting and perforation [45,46]. Histologic examination of an enucleated eye with a corneal ulcer and perforation demonstrated necrotic corneal stroma and infiltration of inflammatory cells, including polymorphonuclear leukocytes and plasma cells in the peripheral corneal stroma [50]. Corneal infiltrates associated with scleritis are also observed in patients with RPC [10,51]. A case of infective keratitis due to corneal intrastromal infiltrate with hypopyon and a case of atypical crystalline keratopathy in a patient with PRC have been reported [20,52].

### 2.5. Eyelid

Lid edema (Figure 4B) is reportedly observed in approximately 8% of patients with RPC [10] and occurs in association with orbital inflammation or independently [10,24,26,29,53]. Retraction and ptosis of the eyelid have also been observed [10]. 

### 2.6. Lens

Posterior subcapsular cataracts are frequently observed, presumably due to prolonged intraocular inflammation and/or the consequence of systemic corticosteroid therapy [2,10].

### 2.7. Retina 

**Figure 5:**
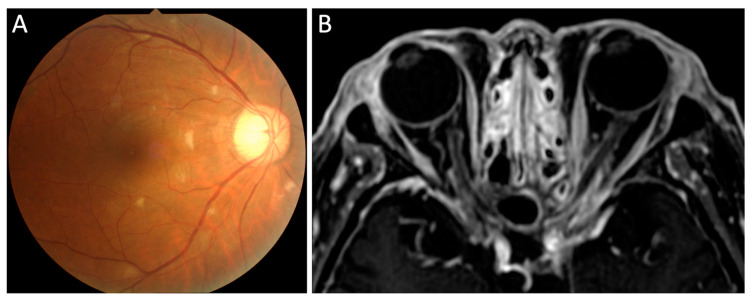
Retinopathy and optic perineuritis in patients with RPC. Fundus photograph shows cotton-wool spots in the retina (**A**). Gadolinium-enhanced T1-weighted MRI with fat suppression shows the “tram-track” enhancement of the left optic nerve sheath (**B**).

In a study by Isaak et al., 8% of RPC patients showed retinopathy consisting of cotton-wool spots (Figure 5A), retinal hemorrhage, and microaneurysms [10]. Retinal vascular occlusion, including central or branched retinal vein occlusion associated with retinal vasculitis, was also observed. Cases with retinal vasculitis, retinal pigment epithelium defects, exudative retinal detachment, and retinal artery occlusion were also reported [19,21,54,55,56]. Cystoid macular edema (Figure 3D) was observed in patients with uveitis.

### 2.8. Optic Nerve and Other Cranial Nerve

Optic neuropathy is a rare ocular complication, although it is the most common cranial nerve disorder associated with RPC. Optic neuropathy includes optic neuritis, papilledema, ischemic optic neuropathy, and optic perineuritis [10,23,27,57,58,59,60,61]. Ischemic optic neuropathy may be induced by systemic vasculitis. We have reported two cases of optic perineuritis [23,27]. One patient presented with recurrent optic perineuritis as the first manifestation of RPC [23]. Optic perineuritis (Figure 5B) occurred sequentially after oculomotor nerve palsy, scleritis, and retinitis in another case [27]. 

Other cranial nerve involvements reportedly include oculomotor and abducens nerve palsies, as well as trigeminal neuralgia [10,22,26,27,62,63]. Oculomotor and abducens nerve palsies result in extraocular muscle palsies and can also be the initial manifestation of RPC [22]. Cao et al. analyzed patients with RPC with central nervous system involvement. Of the 25 patients, two had optic nerve involvement, two had oculomotor nerve involvement, one had trigeminal nerve involvement, and one had abducens nerve involvement [12].

### 2.9. Orbit and Miscellaneous

Although it is uncommon, idiopathic orbital inflammation and inflammatory pseudotumor have been observed in RPC, leading to proptosis, periorbital lid edema, eye pain, and restriction of extraocular movements [10,24,25,64,65,66,67,68,69,70,71,72,73]. Proptosis with chemosis due to orbital inflammation may be the first manifestation of RPC [25]. Orbital inflammation sometimes affects the cranial nerve and induces oculomotor nerve palsy, optic perineuritis, and tumor invasion of the optic nerve [65,67,68]. Although a biopsy may sometimes be difficult due to the lesion location, biopsy of the orbital mass may be helpful to confirm the type of inflammation and exclude malignancy. Tucker et al. examined the histopathology of orbital masses showing reactive lymphoid hyperplasia [43]. Lichauco et al. reported a case of RPC with an orbital mass, and a biopsy of the orbital lesion showed mucosa-associated lymphoid tissue B-cell lymphoma [74]. In addition, a high incidence of hematological malignancies, including leukemia, multiple myeloma, and lymphoma, has been reported in RPC patients [28,75,76,77]. Therefore, biopsy of the orbital mass should be considered when the effectiveness of corticosteroid treatment is insufficient.

Other reported ocular complications associated with RPC include exophthlmos and dacryocystitis.

## 3. Systemic Manifestations 

RPC affects various tissues and manifests with various symptoms. The systemic symptoms of RPC other than ocular symptoms are listed in Table 5. Auricular chondritis is the most common manifestation of RPC, causing bilateral or unilateral auricular pain, redness, and swelling. While approximately 60–90% of RPC patients experience auricular chondritis during the course of the disease (Table 6), only 40% of patients present ear involvement initially [4,10,14]. Prolonged and repeated inflammation of the ear pinna destroys the pinna, resulting in floppy pinna or cauliflower ear [78]. Therefore, ophthalmologists should pay attention to ear swelling and redness in patients with ocular inflammation at the time of the initial examination and during the course of the disease. 

RPC is reportedly associated with many other autoimmune conditions, including systemic vasculitis, rheumatoid arthritis, systemic lupus erythematosus, Behçet’s syndrome, spondylarthritis, and inflammatory bowel disease. In addition, cases of RPC with hematologic diseases, such as myelodysplastic syndromes, lymphoma, or leukemia, have been reported [79,80].

## 4. Laboratory Findings and Diagnosis

Diagnosis of RPC is determined by the clinical manifestations and/or pathologic examination according to the diagnostic criteria. To date, several clinical diagnostic criteria have been proposed. In 1976, McAdam et al. first proposed the diagnostic criteria of RPC [2]: three or more of six clinical features, including auricular chondritis, nonerosive inflammatory polyarthritis, nasal chondritis, respiratory tract chondritis, audio vestibular damage, and ocular inflammation, are needed for diagnosis. Damiani and Levine [3] modified McAdam’s criteria by adding histological confirmation and response to treatment. Michet et al. [4] also proposed diagnostic criteria for clinical symptoms without histological confirmation.

There is no specific laboratory testing for RPC; C-reactive protein levels and erythrocyte sedimentation rate (ESR) are usually elevated, reflecting systemic inflammatory responses. Although the specificity is not very high, anti-type II collagen antibody is detected in the acute phase of RPC, and the serum level of this antibody reportedly correlates with disease severity [81]. Increased levels of anti-matrilin 1 antibody [82] and cartilage oligomeric matrix protein [83] are also reportedly increased in the acute phase of RPC.

**Figure 6:**
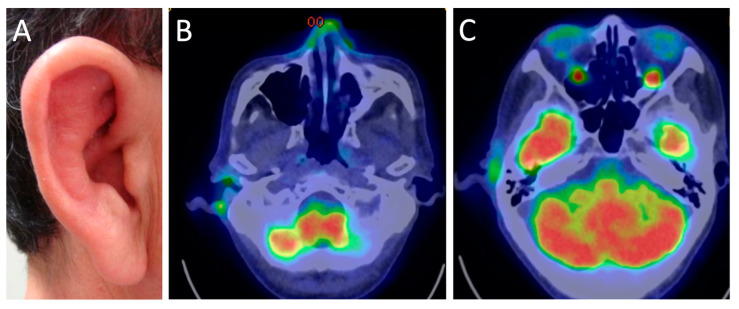
PET/CT images of a patient with right auricular chondritis, nasal chondritis, and bilateral scleritis due to RPC. The right pinna is red and swollen (**A**), and FDG-PET/CT shows high signal intensities (green) in the right auricle, nasal cartilage (**B**), and bilateral sclera (**C**).

Imaging tests, such as computed tomography (CT), magnetic resonance imaging (MRI), 18F-fluorodeoxyglucose-positron emission tomography/CT (FDG-PET/CT), and color Doppler ultrasonography, are useful tools for estimating local inflammation and diagnosing RPC. CT is primarily used for evaluation from the larynx to the subsegmental bronchi. Chest CTs can show airways thickening without a posterior membranous wall and narrowing because of cartilaginous destruction. In particular, expiratory CTs have shown air trapping in the early stages [84]. FDG-PET/CT is an effective imaging modality for detecting all RPC lesions, including auricular, nasal cartilage, larynx and trachea, bronchial, costal, and joints chondritis (Figure 6); FDG-PET/CT can lead to early diagnosis and the evaluation of the disease activity and therapeutic effects [85]. 

**Figure 7:**
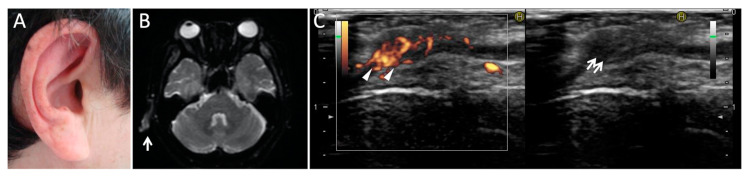
Magnetic resonance imaging and ultrasonography of auricular chondritis in a patient with optic perineuritis due to RPC. The right pinna is red and swollen (**A**) and diffusion-weighted magnetic resonance imaging (**B**) shows signal hyperintensity of the right auricle (arrow). Ultrasonography (**C**) reveals a high-power Doppler signal surrounding the cartilage (arrowheads) and swollen auricular cartilage (arrows).

MRI is also a useful tool for evaluating articular and ear involvement. Perichondrium, chondroepiphysis, and acrophsis are fluid-sensitive sequences of hyperintensity and enhancement after gadolinium administration. RPC with inner-ear involvement shows enhancement of the vestibular area [86]. Diffusion-weighted magnetic resonance imaging can also detect auricular inflammation as a hyperintensity signal in patients with RPC (Figure 7) [87]. 

The clinical implications of ultrasonography of the ear pinna in the diagnosis and monitoring of disease activity of RPC have been reported (Figure 7) [88]. Color Doppler ultrasonography is a convenient, rapid, and noninvasive tool for the estimation of tissue inflammation and blood flow. We have also demonstrated the usefulness of differential diagnosis of giant cell arteritis in patients with ischemic optic neuropathy [89,90]. Therefore, color Doppler ultrasonography of the ear pinna and temporal arteries may be useful for the differential diagnosis of RPC and arteritic ischemic optic neuropathy.

## 5. Treatment

Although there are several review articles about the treatment of RPC [80,91,92], there has been no randomized clinical trial or evidence-based guideline for the treatment of RPC due to the rarity of the disease. In general, topical treatment alone for ocular inflammation in RPC is insufficient in most cases, and systemic treatment with a rheumatologist may be needed. Various anti-inflammatory therapeutic modalities have been reported, including nonsteroidal anti-inflammatory drugs (NSAIDs), corticosteroids, immunosuppressants, and biologics, depending on the severity of the disease. In mild cases, NSAIDs, dapsone, and colchicine are used [79,80,93,94]. Systemic glucocorticoids are needed for severe cases, including ocular inflammation, and usually require long-term oral administration to prevent relapse. Immunosuppressants, including cyclophosphamide, methotrexate, azathioprine, and cyclosporine, are used as second-line options for steroid-intolerant patients or patients needing steroid-sparing [6,9,81]. Recently, various biologics, including infliximab, etanercept, adalimumab, rituximab, anakinra, tocilizumab, and abatacept, have reportedly been used [91,92,95,96,97]. 

Refractory scleritis associated with RPC has been successfully treated with immunosuppressants (cyclosporine, azathioprine, and cyclophosphamide) [35,98,99], infliximab (anti-tumor necrosis factor α antibody) [100,101], or tocilizumab (anti-interleukin-6 receptor antibody) [102,103], in addition to corticosteroids. Surgical interventions may also be required for complications of ocular inflammation, such as cataract, secondary glaucoma, or perforation of the cornea [47,104,105]. 

## 6. Conclusions

Although RPC is a potentially fatal and sight-threatening disease, the rarity of the disease, protean clinical presentations, and wax-and-wane disease courses may lead to missed or delayed diagnosis of RPC [8,9,14]. Early diagnosis of RPC and prompt treatment are critical to prevent RPC-associated complications and death and to improve prognosis. Ocular manifestations also vary, as described in this review. Given the high prevalence of ocular involvement in RPC, to avoid misdiagnosis, physicians should be suspicious of RPC when they see patients with recurrent ocular inflammatory conditions with various systemic symptoms.

## Figures and Tables

**Table 1:** Ocular manifestations in patients with RPC.

Eye Lid	Lid Edema, Ptosis, Tarsitis, Horner’s Syndrome
Conjunctiva	Conjunctivitis, keratoconjunctivitis sicca
Cornea	Ulceration, peripheral thinning, infiltrates, perforation
Sclera	Scleritis, episcleritis, scleromalacia
Uvea	Uveitis
Lens	Cataract
Retina	Retinopathy, retinal vein and artery occlusion, retinal detachment, Retinal vasculitis, retinal pigment epithelium defects, Cystoid macular edema, choroiditis
Optic nerve	Optic neuritis, optic perineuritis, ischemic optic neuropathy, Papilledema
Orbit	Orbital inflammation, proptosis
Others	Dacryocystitis, extraocular muscle palsies

**Table 2:** Incidence of ocular involvement in cases of RPC in a large case series.

	Number of Patients	Country	Study Design	Ocular Inflammation	
				Initial	Cumulative
Cao et al., 2021 [12]	181	China	Retrospective		38%
Chen et al., 2021 [13]	295	China	Retrospective		46%
Zhang et al., 2021 [14]	126	China	Retrospective	20%	27%
Ferrada et al., 2018 [15]	304	International	Survey	40%	53%
Dion et al., 2016 [16]	142	France	Retrospective		56%
Lin et al., 2016 [8]	158	China	Retrospective	11%	44%
Hazra et al., 2015 [6]	50	U.K.	Retrospective		20%
Oka et al., 2014 [17]	239	Japan	Survey	9%	46%
Mathew et al., 2012 [7]	43	USA	Retrospective		57%
Trentham et al.,1998 [9]	66	USA	Retrospective	24%	57%
Zeuner et al., 1997 [18]	62	German	Survey	32%	50%
Michet et al., 1986 [4]	112	USA	Retrospective	19%	51%
McAdam et al., 1976 [2]	23	USA	Retrospective	9%	61%

**Table 3:** Incidence of major ocular manifestations in four large case series.

	McAdam et al., 1976 [2]	Issak et al., 1986 [10]	Zeuner et al., 1997 [18]	Oka et al., 2014 [17]
Number of Patients	159	112	62	239
Total ocular symptoms	65%	51%	50%	46%
Scleritis/Episcleritis	41%	47%	23%	26%
Conjunctivitis	35%	5%	24%	15%
Uveitis	26%	9%	3%	11%
Corneal infiltrate/thinning	-	7%	-	-
Retinopathy	-	8%	-	-
Optic neuropathy/neuritis	4%	6%	-	-
Eyelid edema	-	8%	-	-
Orbital inflammation	-	5%	-	
Extraocular muscle palsy	4%	4%	-	

**Table 4:** Types of scleritis in previous case series.

	Number of Patients	Episcleritis	Anterior			Posterior
			Diffuse	Nodular	Necrotizing	
Hoang et al., 1990 [35]	11	0	5 (45%)	3 (27%)	3 (27%)	0
Sainz-de-la-Maza et al., 2016 [32]	13	0	10 (77%)	0	3 (23%)	0
Yang et al., 2019 [34]	10	0	9 (90%)	1 (10%)	0	0
Tanaka et al., 2019 [33]	9	1 (11%)	6 (67%)	0	0	2 (22%)

**Table 5:** Clinical systemic manifestation of RPC.

Involvement	Symptoms/Manifestations
Ear	Auricular chondritis, hearing loss, tinnitus, serous otitis media, Vestibular dysfunction (vertigo, ataxia, nausea, vomiting)
Nose	Nasal chondritis, saddle nose deformity, rhinorrhea, epistaxis
Respiratory	Hoarseness, cough, aphonia, dyspnea, wheezing inspiratory stridor, Laryngotracheal stricture and collapse
Renal	Elevation of creatinine, microhematuria, proteinuria, necrotizing glomerulonephritis, Glomerulosclerosis, IgA nephropathy, tubulointerstitial nephritis
Musculoskeletal	Arthritis, costochondral cartilage tenderness, flail chest, dislocation
Cardiovascular	Valvular heart disease, aneurysm, pericarditis, vasculitis, Coronary heart disease, tachycardia, atrioventricular block
Skin	Urticaria, purpura, oral aphthosis, angioedema, erythema multiforme, Erythema nodosum, livedo reticularis, panniculitis, superficial phlebitis, dermatomyositis
Neurologic	Headaches, cranial neuropathies, encephalopathies, Seizures, hemiplegia, ataxia
General	Fever, Fatigue, weight loss, night sweat, swelling of lymph nodes

**Table 6:** Incidence of major systemic manifestations of RPC in large case series.

Symptoms	Zhang et al., 2021 [14]	Dion et al., 2016 [16]	Lin et al., 2016 [8]	Oka et al., 2014 [17]	Zeuner et al., 1997 [18]	Michet et al., 1986 [4]	McAdam et al., 1976 [2]
Number of Patients	126	142	158	239	62	112	23
Auricular chondritis	60%	89%	68%	78%	94%	85%	91%
Hearing loss	12%	27%	25%	22%	19%	26%	61%
Vestibular dysfunction	NA	20%	18%	27%	23%	13%	57%
Nasal chondritis	18%	63%	54%	39%	57%	54%	70%
Laryngotracheal	48%	50%	69%	50%	31%	48%	48%
Renal involvement	0%	0%	2.5%	6.7%	6.5%	14%	NA
Arthritis	18%	69%	56%	39%	53%	52%	83%
Valvulopathy	0.8%	22%	1.9%	2.1%	0%	6.3%	17%
Skin involvement	1.6%	28%	46%	13%	24%	28%	39%
CNS involvement	4.8%	7.7%	12%	9.6%	9.7%	NA	NA

CNS, central nervous system. NA, not available.

## Data Availability

Not applicable.
